# A Comparison of Safety, Health, and Well-Being Risk Factors Across Five Occupational Samples

**DOI:** 10.3389/fpubh.2021.614725

**Published:** 2021-02-05

**Authors:** Ginger C. Hanson, Anjali Rameshbabu, Todd E. Bodner, Leslie B. Hammer, Diane S. Rohlman, Ryan Olson, Brad Wipfli, Kerry Kuehl, Nancy A. Perrin, Lindsey Alley, Allison Schue, Sharon V. Thompson, Megan Parish

**Affiliations:** ^1^School of Nursing, Johns Hopkins University, Baltimore, MD, United States; ^2^Oregon Healthy Workforce Center, Oregon Institute of Occupational Health Sciences, Oregon Health & Science University, Portland, OR, United States; ^3^OHSU-PSU School of Public Health, Portland State University, Portland, OR, United States; ^4^Occupational and Environmental Health, University of Iowa, Iowa City, IA, United States; ^5^School of Medicine, Oregon Health & Science University, Portland, OR, United States; ^6^College of Osteopathic Medicine, Western University of Health Sciences, Lebanon, OR, United States; ^7^Division of Nutritional Sciences, University of Illinois at Urbana-Champaign, Urbana, IL, United States; ^8^Confluence Health, Wenatchee, WA, United States

**Keywords:** health promotion, health behaviors, occupational safety, health, well-being

## Abstract

**Objective:** The aim of this study was to present safety, health and well-being profiles of workers within five occupations: call center work (*N* = 139), corrections (*N* = 85), construction (*N* = 348), homecare (*N* = 149), and parks and recreation (*N* = 178).

**Methods:** Baseline data from the Data Repository of Oregon's Healthy Workforce Center were used. Measures were compared with clinical healthcare guidelines and national norms.

**Results:** The prevalence of health and safety risks for adults was as follows: overweight (83.2%), high blood pressure (16.4%), injury causing lost work (9.9%), and reported pain (47.0%). Young workers were least likely to report adequate sleep (46.6%). Construction workers reported the highest rate of smoking (20.7%). All of the adult workers reported significantly lower general health than the general population.

**Conclusion:** The number of workers experiencing poor safety, health and well-being outcomes suggest the need for improved working conditions.

## Introduction

There is growing awareness in the literature that providing a healthy labor force requires integrated consideration of each workplace's impact on employees' safety, health, and well-being (1). This relationship between work and well-being is further impacted by changing trends within the American workforce as well as the nature of work. For example, there is a growing number of working older adults. It is estimated that by 2024, the employment rate of workers 65–74 years is projected to grow by 55% and that of workers 75 years and older is expected to grow by 86% (2). Further, while physically hazardous jobs with high risk of injury and illness continue to exist, jobs that increase the risk of chronic illness are becoming increasingly prevalent as employees remain inactive for long hours, experience high job stress and burnout, and face greater job insecurity and occupational health disparities.

Moreover, the prevalence of preventable chronic health conditions across all age groups is increasing (3). About 60% of the U.S. population suffers from at least one chronic health condition (4), and healthcare costs associated with these conditions account for 75% of healthcare spending (5). Modifiable exposures and health risk factors such as stress, physical inactivity, and obesity account for ~26% of employer healthcare costs, at $761 per employee (6).

Occupational injuries in the U.S. workforce continue to be a concern, with 3.2 cases per 100 full-time workers in the private sector and 5.0 per 100 in the public sector in 2014 (7). Furthermore, there are bi-directional interactions of safety and health. For example, workers with obesity who experience workplace injuries experience 80.0% greater working time loss and incur 81.4% higher costs than workers without obesity (8). Another example can be found among commercial truck drivers, where drivers with untreated sleep apnea have a five-fold risk of a serious crash (9). A holistic intervention approach that targets workplace safety, health, and worker well-being can curtail costs from largely preventable workplace injuries and chronic illnesses.

To this end, in 2011, the National Institute for Occupational Safety and Health (NIOSH) launched *Total Worker Health*® (TWH), an approach that recognizes that work is a key determinant of one's health and well-being. This approach prioritizes a hazard-free work environment and emphasizes integrated interventions that collectively target worker safety, health, and well-being. TWH is defined as policies, programs, and practices that integrate protection from work-related safety/health hazards with promotion of injury and illness prevention efforts to advance worker well-being (10, 11). As part of this effort, NIOSH funded the Total Worker Health Centers of Excellence (12), one of which is the Oregon Healthy Workforce Center (OHWC) (13).

An integrated effort first requires monitoring of the safety, health, and well-being risk factors at employee and organizational levels; doing so will help us identify targets for change. At OHWC, we have created a repository of data collected via a set of common measures used across multiple projects, with the goal of comparing safety and health data of participants from various industry sectors. This fairly novel approach has the potential to improve the quality and utility of occupational health research by facilitating stronger comparisons across populations.

### Common Measures Approach vs. Meta-Analyses

Occupational health meta-analyses have helped identify relationships between workplace risk factors and employee health outcomes, including correlations between job strain and leisure-time physical inactivity (14), and work stress and tobacco smoking (15). Although such meta-analyses can be powerful, measuring the same construct using different survey items on different scales of measurement, can add error to the conclusions. Meta-analyses can overcome differences in measurement tools by using effect sizes that serve as a standardized measure. Although this approach works well when examining the relationship between different variables, it cannot be applied when comparing single-risk factors across different occupational groups. Using the same measure across studies is a way to increase the precision of the measurement by reducing variability due to the way the survey items are measured.

A common measures approach has multiple advantages. We can utilize the same measures across different study populations to benchmark comparisons of the data. Further, given that there are 19,256 unique industry sectors in the U.S. workforce (16), standardizing the safety and health measures across sectors within occupational safety and health intervention studies allows us to test the effectiveness of program components within and between populations. In turn, this will expedite the process of translating and disseminating interventions to diverse work settings (17). The goal to increase standardization in measurement is consistent with NIH's funding to develop and promote PROMIS®, a set of standard measures that assess physical, social, and mental health among adults and children (18).

### Comparing Common Outcomes Across Studies vs. Population-Based Studies

Most studies examining health risks have focused on a specific occupational setting or have used random sampling to estimate the overall population risk (19–23). Although both of these methods make important contributions to understanding the relationship between work and health, both methods leave some gaps. For example, general population studies typically include working and non-working individuals. Further, information about occupations may be limited to broad categories such as white-collar vs. blue-collar occupations (22). All of the population-based studies we found were conducted among working populations outside of the United States, often in European countries where governments sponsor recurring studies on working conditions (15, 20, 21, 24). Generalizations to the U.S. are limited due to possible differences in national policies, work experiences, organizational culture, population health status, and occupational health risk factors. Moreover, large population studies are costly and are conducted only periodically. For example, the European Working Conditions Surveys are collected every 5 years and focus on work-related exposures, not on the impact of work on individual health behaviors (21).

A common measures approach has unique strengths and weaknesses. It can be a powerful research strategy to surveil the safety and health of the workforce, make comparisons between occupations, and inform intervention strategies that are best suited within and across workplace settings. A challenge of the common measures approach is that it can involve a high degree of coordination and buy-in from separate collaborators. However, the advantage is the ability to use individual data on the same scale of measurement to make direct comparisons. This approach may be less expensive and resource-intensive than larger population-based studies. The advantage of a less expensive approach is that it can be done more frequently or fill in the gaps between costly population-based occupational groups. These “grass roots” efforts can be especially helpful in continuously monitor the safety and health of workers as the nature of the work continues to evolve with changes in technology, shifts in economic policies, and other changing factors in the landscape of work.

We found one other study that uses this common measures approach: Community Interventions for Health (CIH)—a collaboration that seeks to understand the impact of health behavior interventions on health outcomes in developing countries (25). Each country agrees to use a core set of measures designed in a way that adds culturally relevant examples and appropriate items. This approach enables CIH to assemble large datasets from multiple countries and highlight the relationships that are common across different countries (26–28).

The OHWC Common Measures Data Repository currently includes data from five separate studies, and we have compared the safety, health, and well-being outcomes of working populations across different occupations. OHWC presents collective and unique profiles of these worker groups: call center workers, corrections officers, construction workers, homecare workers, and parks and recreation workers. Each work setting includes unique hazards and risk factors, and physical and psychological demands (29). For example, homecare workers often receive little safety training or health benefits, work primarily alone, and are responsible for lifting and moving their consumer-employers multiple times per day (30–32). Construction workers also face considerable physical demands, but have a great deal more supervision and adhere to rigid schedules, making them particularly susceptible to issues regarding work-family conflicts and psychological stress (33).

## Methods

### Measures

Baseline data were gathered from five studies funded by NIOSH. A standardized set of measures was agreed upon prior to data collection for each study. From this set, individual study teams selected the measures that best fit their needs. Thus, not every sample reported data on every variable. For purposes of our study, we chose measures of safety (injuries), health [pain, body mass index (BMI), blood pressure], health behaviors (smoking, sleep, exercise), and well-being (health status) used by at least three of our studies. Where possible we computed these variables so that they could be compared with clinical healthcare recommendations or national norms. Additionally, biomarker assessment was conducted by a trained research assistant unless otherwise indicated.

#### Injuries

Injuries were measured with a single item: “In the last 6-months, if you had 1 or more injuries at work that required you to miss work on following shifts, how many total work days did you miss?” Responses were coded 0 (No missed days) or 1 (Yes, 1 or more missed days). The 6-month timeframe was chosen because research indicates that participant recollection of medical events are less accurate for 1-year than for 1-month (34) however, injuries are rare and thus 1-month was not ideal. Given this 6-months seemed a reasonable compromise between exposure and accuracy.

#### Pain

Musculoskeletal pain that interfered with normal activities was measured with four items adapted from the Standardized Nordic Questionnaires for the Analysis of Musculoskeletal Symptoms (35). The items asked how often in the last 3 months pain interfered with normal activities at work or at home. The following body areas were included: neck/shoulder, lower back, wrist or forearm, and lower extremities. For the present study, participants were coded as 0 if they answered “not at all” to all questions and 1 if they reported any interference with work on any of the four items.

#### Health Status

Health status was measured using the SF12v2, which contains 12 survey items measuring eight subscales: general health, physical functioning, role physical, role emotional (i.e., ability to perform role-related responsibilities due to emotional or physical health issues) bodily pain, mental health, vitality, and social functioning. The scale has been validated for use in general U.S. populations, in 10 other countries, and in populations of individuals with a variety of health conditions. Extensive information about the reliability and validity of the SF12v2 can be found in the SF12v2 instruction manual (36). Scores were normed using means and standard deviations from a representative sample of the general U.S. population described in the Participants section of the present paper. Per instructions in the manual, *z*-scores were computed by subtracting the provided mean for each subscale from the general U.S. sample and dividing by the provided standard deviation for the subscale from the general U.S. sample. Following the instructions in the manual *t*-score transformations were computed by adding 50 and multiplying by 10. This facilitated a comparison to that national representative sample with a mean of 50 and a standard deviation of 10.

#### BMI

BMI and cut-offs for overweight and obesity were calculated based on CDC guidelines (37). Participants were weighed with clothes on, pockets emptied, and no shoes, belts or heavy jewelry/watches, etc. For adults, BMI was calculated using the standard formula: weight (kg)/height (m)^2^. For workers under 18y, BMI was computed based on sex-specific age growth charts. For both groups, individuals were coded as overweight if they had a BMI of 25.0–29.9 and obese if they had a BMI of 30.0 or greater.

#### Blood Pressure

Blood pressure was taken after 3 min rest followed by 3 measurements, each 1 min apart; then we took the average of those three measurements. Blood pressure cut-offs for pre-hypertension and hypertension were based on NIH National Heart, Lung, and Blood Institute (NHLBI) recommendations (38). Cases were coded as pre-hypertensive if they had a systolic blood pressure of 120–139 mm Hg or a diastolic blood pressure of 80–89 mm Hg, and as hypertensive if they had a systolic blood pressure of ≥140 or a diastolic blood pressure of ≥90 mm Hg. We did not inquire as to whether workers were participating in anti-hypertensive treatment at the time of data collection.

#### Smoking

Participants were asked: “In the past 7 days, have you smoked any cigarettes?” Responses were coded 0 (no) or 1 (yes). This is consistent with the U.S. Department of Health and Human Services' initiative to end the tobacco epidemic (39).

#### Sleep

Sleep was measured using two items from the Pittsburgh Sleep Quality Index (40) to compute time spent in bed. Minimum guidelines for sleep were adopted from the CDC (41). Adults were coded as meeting the minimum guidelines if they got at least 7 h of sleep; young workers were coded as meeting the minimum guideline if they got at least 9 h of sleep per night.

#### Exercise

For all of the adult participants, exercise was coded as “yes” if the participant reported engaging in moderate or vigorous exercise for 30 min on 5 or more days per week [per CDC recommendations (42)] and “no” if they did not. In the young worker sample, participants were not asked about intensity (“moderate/vigorous”).

### Participants

#### Call Center Workers

Participants included 139 employees from two customer service call centers. There are ~29,000 customer service employees in Oregon (43). Employees were recruited by study advertisements and completed all study activities during work hours. Participants received a $25 gift card for completing the study. Data were collected in the summer through fall of 2015. All study procedures were approved by Oregon Health & Science University (OHSU) IRB #0753.

#### Correction Officers

Participants in the first study included 85 corrections officers from four Oregon Department of Corrections institutions. Oregon employs ~2,300 correction officers in 14 state prisons (44). Prior to recruiting participants, permission was granted by the Superintendent of each institution. Participants were full-time security staff at the institutions. Data were collected between June 2011 through May 2013. All study procedures were reviewed and approved by OHSU IRB #7925.

#### Construction Workers

Participants in the second study included 349 construction workers from two public works agencies with a total of 520 construction workers, giving us a response rate of 67.12%. There are ~80,000 construction workers in Oregon (43). The results from the main study are published in the article cited here (45). Data were collected on company time in the summer of 2012. Participants were provided a $25 gift card for their participation. All study procedures were reviewed and approved by Portland State University IRB #111884.

#### Homecare Workers

Participants in the third study included 148 Oregon homecare workers recruited from the population of caregivers enrolled in a publicly funded home care system overseen by the Oregon Home Care Commission (31). There were ~12,000 homecare workers registered with the OHCC in the spring of 2013 when we collected these data (46). Within this system, caregivers work as independent contractors and are hired directly by “consumer-employers” who qualify for Medicaid-funded in-home services. With the assistance of the Service Employees International Union SEIU and the Commission, workers were recruited in-person at training classes, but also through emails, mailed fliers, and referrals. All study procedures were reviewed and approved by OHSU IRB #5473. The results of the main study are published in the article cited here (31).

#### Parks and Recreation Workers

In the summer of 2013, we sent emails to 436 young workers (14–24 years of age) from a city parks and recreation department who were seasonal summer employees. Throughout the results and discussion we refer to this sample of 14–24 year olds as young workers and our other samples of workers aged 25 and older as adult workers. Of those invited to participate 178 completed baseline surveys, a response rate of 40.83%. Results from the main study are published in the article referenced here (47). There are about 1,800 parks and recreation workers in the state of Oregon (43). Participants were recruited during new hire orientation; parental consent letters were distributed to minors. No biomarkers were assessed in this study. All study materials and procedures were approved by OHSU IRB #0753.

#### U.S. General Population Norming Means and SD

The means and SD for norming the scores for comparison to the U.S. general population are in the SF12v2 scoring manual (36). These data are from the 1998 National Survey of Functional Health Status (NSFHS), conducted from October to December 1998 by the National Research Corporation (NRC). Surveys were mailed to randomly selected members of the National Family Opinion (NFO) panel; 7,069 participants responded (overall response rate: 67.8%). The population contained both working and non-working adults. Sampling weights were applied to adjust the sample to match the age, gender, and age-by-gender distribution of the 1998 census.

### Analyses

Descriptive statistics, frequencies, means, and standard deviations were computed to create profiles for these participating workers. One-sample *t*-tests were used to test whether the normed scores from our participants on the SF-12 subscales were statistically different from a nationally representative sample, with a mean of 50 for all subscales. Alpha was set at *p* = 0.05 for a two-tailed test for determining statistical significance.

## Results

### Demographics and Work Characteristics

A comparison of the demographics and work characteristics of the five samples in Table 1.

**Table 1:** OHWC descriptive statistics, demographics, and work characteristics.

	**Call center workers**		**Corrections officers**		**Construction workers**		**Homecare workers**		**Parks and recreation workers**	
	***N***	**M ± SD**	***N***	**M ± SD**	***N***	**M ± SD**	***N***	**M ± SD**	***N***	**M ± SD**
Age	139	38.26 ± 10.47	83	42.66 ± 10.05	347	44.48 ± 9.56	148	51.70 ± 13.19	178	17.98 ± 2.24
Hours/week	N/A	N/A	79	42.11 ± 4.01	324	41.77 ± 6.27	129	24.01 ± 17.05	N/A	N/A
	***N***	**%**	***N***	**%**	***N***	**%**	***N***	**%**	***N***	**%**
Gender (male)	139	64.8%	84	75.0%	347	89.1%	142	7.7%	178	46.1%
Race	139		76		343		142		178	
White		63.3%		85.5%		77.3%		83.8%		75.8%
Black		11.5%		6.6%		6.7%		0.0%		4.5%
Native		3.6%		2.6%		2.6%		7.7%		2.2%
American										
Asian		2.2%		0.0%		2.6%		2.1%		6.7%
Native		2.2%		0.0%		0.3%		2.1%		0.0%
Hawaiian/Pacific islander										
Multi-racial		5.8%		2.6%		8.5%		4.2%		9.0%
Other		11.5%		2.6%		2.0%		0.0%		1.7%
Hispanic	135	16.3%	77	6.5%	342	2.6%	132	6.1%	178	6.2%
Education	N/A	N/A	79		346		145		178	
Less than HS				0.0%		2.3%		1.4%		48.3%
HS/GED				20.3%		37.3%		33.1%		20.3%
Some college				64.6%		47.7%		40.0%		27.5%
Bachelor's or >				15.2%		12.7%		25.5%		3.9%
Tenure	N/A	N/A	79		347		145		178	
<1 year				0.0%		4.0%		11.7%		35.4%
1–3 years				20.3%		15.3%		30.3%		36.5%
>3 years				79.8%		80.7%		58.0%		28.0%

*For the purpose of this study, we define Hispanic as an individual who identifies as being of Cuban, Puerto Rican, South or Central American, or other Spanish culture of origin regardless of race*.

### Comparison Across Measures of Safety, Health, Health Behaviors, and Well-Being

Table 2 provides an overview of the safety and health profiles for all worker samples. Ten percent of older adult workers (i.e., 65 and above; call center, construction, corrections, and homecare) reported work-related injuries that resulted in missed work during the past 6 months. Such injuries were highest among construction workers at 16.2%. Forty-seven percent of adult workers reported experiencing pain in the last 6 months that interfered with normal activities. More than 70% of all participants were overweight or obese. In the young worker sample, just over 21% were overweight or obese. Conversely, 83.2% of older adult workers were overweight or obese. Among the adult participants, 16.4% had high blood pressure (HBP) and 41.0% were pre-hypertensive. Approximately 15% of all workers reported smoking in the last week/month. Smoking was lowest among young workers employed by parks and recreation department (4.5%) and highest among construction workers (20.7%). Approximately 60% of all workers reported getting sufficient sleep; as recommended by NIH. Sleep sufficiency was lowest in the young worker sample (46.6%) for whom more sleep is recommended. Only 35% of the workers were getting 150 min of moderate to vigorous physical activity per week as recommended by the CDC. Young workers were more likely to meet exercise guidelines, yet even in this sample, just over 50% met the guidelines. Physical activity was lowest among corrections officers and homecare workers, at just over 20%.

**Table 2:** OHWC descriptive statistics for health, safety, well-being, and health behaviors.

	**Call center workers**		**Correction officers**		**Constructions workers**		**Homecare workers**		**Parks and recreation workers**		**Combined sample**	
	***N***	**%**	***N***	**%**	***N***	**%**	***N***	**%**	***N***	**%**	***N***	**%**
**Safety**												
Work injury that required lost work days	139	2.9%	79	5.1%	346	16.2%	147	4.1	N/A	N/A	572	9.9%
Pain that interfered with normal activities	123	6.3%	79	44.3%	341	56.0%	148	61.6%	N/A	N/A	566	47.0%
**Health**												
Smoking in past week	138	16.7%^a^	79	12.7%	348	20.7%	147	16.3%^a^	178	4.5%	752	15.4%
Recommended hours sleep (teens 9+ h; adults 7+ h)	139	79.3%	77	56.4%	339	60.2%	N/A	N/A	178	46.6%	594	60.1%
Moderate/vigorous exercise for 30 min for 5 or more days per week	139	43.1%	79	21.5%	346	32.1%	145	22.8%	178	50.6%^b^	748	35.1%
BMI	138		83		335		148		178		744	
Overweight		15.9%		28.9%		31.3%		24.3%		13.5%		23.9%
Obese		60.9%		63.9%		54.0%		54.7%		7.9%		46.8%
Blood Pressure	130		82		336		147	34.7%	N/A	N/A	565	
Pre-hypertension		33.8%		53.7%		51.2%		17.0%				41.0%
Hypertension		10.0%		19.5%		25.3%						16.4%
	***N***	**M ± SD**	***N***	**M ± SD**	***N***	**M ± SD**	***N***	**M ± SD**	***N***	**M ± SD**	***N***	**M ± SD**
**SF-12**												
General health	N/A	N/A	79	41.14 ± 10.13^***^	345	45.60 ± 9.70^***^	147	44.36 ± 10.25^***^	178	51.18 ± 9.00	749	46.21 ± 10.15^***^
Physical functioning	N/A	N/A	78	52.61 ± 6.44^**^	343	53.09 ± 6.98^***^	146	46.35 ± 9.97^***^	178	53.47 ± 7.37^***^	745	51.81 ± 8.14^***^
Role physical	N/A	N/A	76	52.39 ± 6.75^**^	N/A	N/A	145	46.98 ± 9.97^***^	178	54.33 ± 5.47^***^	399	51.29 ± 8.31^**^
Role emotional	N/A	N/A	78	48.34 ± 9.38	N/A	N/A	144	47.81 ± 9.98^**^	178	49.80 ± 8.87	400	48.80 ± 9.40^*^
Bodily Pain	N/A	N/A	79	49.19 ± 9.80	348	46.14 ± 11.16^***^	145	45.92 ± 10.35^***^	178	53.95 ± 6.32^***^	750	48.27 ± 10.42^***^
Mental health	N/A	N/A	79	46.25 ± 10.33^**^	344	47.94 ± 10.10^***^	147	47.54 ± 10.66^**^	178	49.85 ± 9.20	748	48.14 ± 10.07^***^
Vitality	N/A	N/A	79	48.13 ± 9.32	344	50.15 ± 9.23	147	48.64 ± 10.79	178	52.50 ± 8.50^***^	748	50.20 ± 9.50
Social functioning	N/A	N/A	79	46.34 ± 11.49^**^	344	49.23 ± 10.09	147	47.43 ± 10.26^**^	178	50.61 ± 8.00	748	48.90 ± 9.90^**^

*N in this table represent the number of participants in each sample who answered each question. % in this table indicated the percent of participants who answered in the affirmative out of those who answered the question for each sample*.

a *Homecare workers and call center workers asked about smoking in the last month*.

b *Parks and recreation workers were not asked about the intensity of their exercise*.

*Scores for each of the sub-scales of the SF-12 were standardized using the means and SDs of the nationally representative sample and converted to t-scores to be comparable to the nationally representative sample with a mean = 50 and a SD = 10*.

*** p < 0.001 and

** *p < 0.010*.

One-sample *t*-tests indicated that construction workers and homecare workers reported being pain-free significantly less often than the U.S. general population (*p* < 0.001); young parks and recreation workers were significantly more pain free (*p* < 0.001). All four of the adult samples had significantly poorer general health (*p* < 0.001) than a nationally representative sample. No evidence could be found that the general health of young workers was significantly different from that of a nationally representative sample. The homecare workers, who were also our oldest sample, had significantly poorer physical functioning than a nationally representative sample (*p* < 0.001). However, all of the other occupational samples had significantly better physical functioning (*p* < 0.010_all 4 samples_). Homecare workers scored significantly lower than the nationally representative sample in both role physical and role emotional (*p* < 0.010); that is, they reported feeling limited in their ability to perform role-related responsibilities due to emotional or physical health issues. The other worker groups were significantly healthier than the U.S. general population on role functioning (*p* < 0.010), but not statistically different from the U.S. general population on role emotional. All of our adult samples reported poorer mental health than the U.S. general population (*p* < 0.010). The parks and recreations workers scored significantly higher on vitality than the US general population (*p* < 0.001). We found no difference between any of the adult samples and the U.S. general population on vitality. Corrections officers and homecare workers scored significantly lower than the general US population on social functioning (*p* < 0.010).

## Discussion

### Overview of Findings

Our findings point to a workforce with both health and safety concerns. With regard to safety, 11% of adult workers reported work-related injuries that resulted in missed work and 47% were experiencing pain that interfered with normal activities. Further, many workers in our studies are at risk for chronic health conditions. Over 70% of the overall sample was overweight or obese and 57% of older adult workers were hypertensive or pre-hypertensive. Our findings show that working populations such as those in our studies can benefit from a Total Worker Health approach that targets factors that can improve health, safety, health behaviors, and well-being.

### Role of the Work Environment on Safety and Health Outcomes

Studies at the Oregon Healthy Workforce Center have found that while individual behaviors play a role in worker health, safety, and well-being, the workplace environment can also have a large impact, such as access to safety equipment, access to healthy foods, reasonable working hours and breaks, access to opportunities to engage in physical activities at or near work (48–51). In addition, workers who are stressed or injured at work may engage in unhealthy behaviors such as poor diet, lack of physical activity, lack of sleep, and substance abuse, which in turn can contribute to further injuries or chronic health conditions such as obesity or HBP (52, 53). Our findings suggest that there is much need to study and improve working conditions for these occupational groups, with the goal of promoting health, safety, and well-being. Specifically, organizations should influence employee lifestyles through structural changes to the design of work and working conditions that would facilitate engaging in these activities, along with programs that target individual motivation and participation.

In our study, there was a high rate of pain reported among workers in corrections, construction and homecare. Population-based studies indicate that levels of musculoskeletal pain in adults range from 6 to 55% (19, 54). In a large random sample of working adults from one UK region, the prevalence of adults with pain in upper limbs and neck was 50.5%. This UK region had a large percentage of manufacturing workers; however, only 13% reported pain that interfered with functioning. In a large random sample of people from Sweden, 55% of the population perceived consistent pain for three 3 months or more (54). This sample consisted of residents from two regions of the country: one with a high percentage of industrial manufacturing and blue-collar workers and the other with a high percentage of fishing and agricultural workers. Factors found to be associated with musculoskeletal pain included the following: repetitive lifting of heavy objects, prolonged neck bending, working with arms at shoulder height or higher, low job control, low supervisor support, blue-collar occupations, and female gender. Growing evidence suggests that work-related injuries play a part in the opioid epidemic (55, 56). Occupations that require a high degree of manual labor such as construction show a higher likelihood that a worker will develop a dependency on prescription opioids (55).

All of our adult samples had lower levels of mental health than the general US population. Workplace factors associated in the literature with decreased mental health include: high job strain—which is a combination of high demands and low discretionary control over work—low social support at work, effort-reward imbalance, shift work (especially night shift), and long work hours (20, 57–61). Organizational interventions to prioritize mental health by reducing sources of job stress and providing access to employee-assistance programs such as confidential counseling are critical. Similarly, increasing job control may help to decrease stress, improve work-life balance, thereby reducing the risk for stress-related outcomes such as hypertension.

### Occupational Differences

A crucial component in identifying cross-population factors related to risks and general wellness at the occupation-level lies in comprehensively understanding the distinct challenges, contexts, and profiles of the workers within each setting (62). Differences between samples could be evidence of structural barriers in workplaces that do not prioritize safety and health behaviors. Research has demonstrated that aspects of the physical environment or nature of work impact safety and health behaviors and related outcomes. For example, at a public health level, the following are related to greater participation in physical activity: accessibility of fitness facilities, the presence of sidewalks, and low-traffic (48). In the work environment, examples of facilitators of physical activity could include pedal stands, having proper work breaks, and safe spaces to walk at work.

Homecare workers had poorer health across several measures compared to the other occupational groups; they also reported greater pain, poorer physical functioning, and role functioning than the U.S. general population. Our previous qualitative research indicated that these homecare workers, who were employed by the consumers or their families, reported low support for safety (32). In an institutional care organization, lifting would be done by a group of workers whereas homecare workers must often do this lifting alone. Because homecare workers are dependent on their consumer and the consumer's case manager to request safety equipment, the process is often unclear for the worker. They also reported poorer well-being as indicated by lower emotional and social functioning than the nationally representative sample. In our previous work we found that homecare workers also reported feeling socially isolated, having almost no contact with co-workers other than during training sessions. This isolation could contribute to lower well-being among homecare workers. These are some aspects of the work environment that could be targeted to decrease injuries and pain, and improve well-being.

Construction workers had the highest rate of injuries and, like homecare workers, reported a high degree of pain interfering with normal activities. Of all the occupational groups, construction workers had among the highest occupational exposure to posture-related risk factors for injury (21). The vast majority of construction workers were overweight or obese and were pre-hypertensive or hypertensive. Smoking was also more prevalent among construction workers than among the other occupations we assessed. Construction workers would benefit substantially from interventions focused on reducing hazardous exposures and work-related injuries, smoking cessation programs (63), and by training supervisors to better support work-life integration (64), and safety communications (65).

Corrections workers reported less pain than our other samples. They also showed better outcome measures of health (i.e., general health) and well-being (e.g., mental health and social functioning) than the U.S. population in general. They did, however, have among the highest percentage of overweight and pre-hypertension/hypertension of our occupational groups. Further research into how the work environment could be modified to reduce risks of preventable diseases could be particularly useful for these workers.

### Younger and Older Workers

There were a variety of notable differences between the younger and older workers. The older workers generally had poorer general and mental health than the general U.S. population. On the other hand, younger workers were no different than the general U.S. population. Research has indicated that reports of pain increase as workers age (54). We saw evidence of this in our sample: two of the older worker samples (homecare and construction) reported significantly more bodily pain than the general population while the young workers reported significantly less pain than the general population. Young workers scored significantly higher on vitality than the U.S. general population (*p* > 0.001); there was no difference between the adult samples and the U.S. general population on vitality. Younger workers, who need more sleep than older adults, were more likely to report inadequate sleep than older workers. TWH interventions geared toward older adults would include healthy pain management strategies (at the individual level) in combination with addressing important changes to the work environment such as providing tools for safe lifting and preventing worksite risks for injuries and accidents. Although young workers are healthier compared with older workers, they could benefit from interventions to increase sleep and physical activity. Intervening with younger workers to establish prevention strategies that are reinforced through their career could be a worthwhile approach that may help to prevent worsening of health conditions as career paths progress (47).

### Limitations

Our study has some limitations. All samples were chosen to address the main aims of the sub-studies making up the OHWC. These occupational groups are not meant to be representative of the entire national workforce but rather these specific occupational groups within Oregon. These were convenience samples within single organizations and thus may not be as representative of their respective occupational groups compared to a study using random sampling of all individuals in a certain occupation. The OHWC targets working populations with high burden and need, which should be considered when generalizing our results. When comparing our samples to the national representative sample, we could not match the age or gender of our samples because we did not have the individual data for the national sample. We cannot rule out the influence of other factors beyond working conditions on workers' health, as the data is cross-sectional and we did not measure pre-existing conditions. In addition, more detail on several of our outcomes would allow conclusions that are more precise. For example, we asked about smoking in the past week. We did not ask how long workers had smoked or whether some may have only recently quit. When including common measures across multiple studies that may not be relevant to other aims in is necessary to trade off details for efficiency. Next, all of these data were collected in the State of Oregon. It is possible that regulations in other states or other state-level variables could influence safety and health behaviors and outcomes for workers in similar occupations. In addition, after we began our data collection for these studies, the NIH published PROMIS measures (66–68)—a set of freely available, well-validated measures of various aspects of health, with the objective of standardizing measures across studies. We have adopted these measures for subsequent data collection across projects, but unfortunately, they could not be part of this study. Finally, some measures referenced varying reflective time periods (e.g., smoking a cigarette in the last week vs. last month); thus, direct comparisons on these specific variables should be made with caution. Nonetheless, the Common Measures Data Repository is a promising approach to learning and addressing the unique and shared needs of worker populations across occupations.

### Practical Implications and Conclusions

Growing literature suggests that lifestyle behaviors such as getting adequate sleep, exercising regularly, eating a healthy diet, and not smoking can be influenced by work exposures, conditions, and policies (69). Because adults spend a significant amount of their awake hours at work and because work plays an important role in our lifestyle and well-being, the workplace is an opportune platform from which to address health behaviors and outcomes.

Using a common measures approach to understand occupational safety, health, and well-being outcomes across studies can serve to compare and contrast risks, and highlight avenues for interventions to reduce work-related hazards and promote health and well-being. The findings of our common measures analyses point to the potential benefit of a Total Worker Health approach, in particular, integrated interventions that can decrease work-related risk factors and improve facilitators for pursuing health, safety, and well-being among workers across industries and along the age spectrum. For example, early interventions to reduce risk for injury at work can prevent the experience of pain among older workers, which in turn could improve health and safety behaviors, enhance health outcomes, and overall facilitate long-term quality of life.

## Data Availability Statement

The datasets presented in this article are not readily available because they must be approved by the OHWC Steering Committee. Requests to access the datasets should be directed to Ginger Hanson, ghanson4@jhu.edu.

## Ethics Statement

The studies involving human participants were reviewed and approved by Oregon Health and Science University and Portland State University. Written informed consent to participate in this study was provided by the participants' legal guardian/next of kin.

## Author Contributions

GH, AR, TB, LH, DR, RO, BW, KK, and NP: conception and design of study. GH, AR, TB, LH, DR, RO, BW, KK, NP, ST, and MP: acquisition of data. GH, AR, TB, and NP: analysis and/or interpretation of data. GH, AR, LA, and AS: drafting the manuscript. AR, TB, LH, DR, RO, BW, and KK: revising the manuscript critically for important intellectual content. All authors contributed to the article and approved the submitted version.

## Conflict of Interest

The authors declare that the research was conducted in the absence of any commercial or financial relationships that could be construed as a potential conflict of interest.
